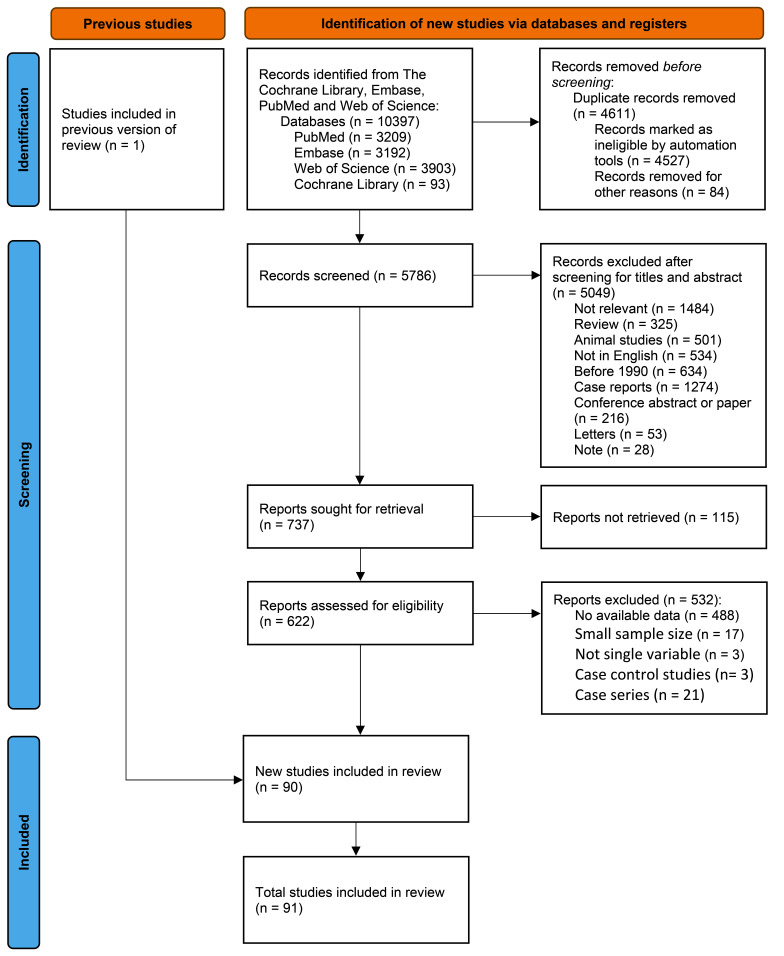# The effect of omentoplasty in various surgical operations: systematic review and meta-analysis

**DOI:** 10.1097/JS9.0000000000001240

**Published:** 2024-03-04

**Authors:** Yaqi Peng, Shan Xiong, Yujin Ding, Limin Xie, Yihang Wang, Ying Mei, Wei Liu, Tuo Deng

**Affiliations:** a National Clinical Research Center for Metabolic Diseases, Department of Metabolism and Endocrinology; b Key Laboratory of Diabetes Immunology, Ministry of Education, Metabolic Syndrome Research Center; c Department of Biliopancreatic Surgery and Bariatric Surgery; d Clinical Immunology Center, The Second Xiangya Hospital of Central South University, Changsha, Hunan, People’s Republic of China

**Keywords:** age, BMI, gastrointestinal surgery, liver surgery, omentoplasty, postoperative complications

## Abstract

**Background:**

Omentoplasty is commonly used in various surgeries. However, its effectiveness is unsure due to lack of convincing data and research. To clarify the impact of omentoplasty on postoperative complications of various procedures, this systematic review and meta-analysis was performed.

**Methods:**

A systematic review of published literatures from four databases: PubMed, Web of Science, Cochrane Library, and Embase before 14 July 2022. The authors primarily included publications on five major surgical operations performed in conjunction with omentoplasty: thoracic surgery, esophageal surgery, gastrointestinal surgery, pelvi-perineal surgery, and liver surgery. The protocol was registered in PROSPERO.

**Results:**

This review included 25 273 patients from 91 studies (*n*=9670 underwent omentoplasty). Omentoplasty was associated with a lower risk of overall complications particularly in gastrointestinal [relative risk (RR) 0.53; 95% CI: 0.39–0.72] and liver surgery (RR 0.54; 95% CI: 0.39–0.74). Omentoplasty reduced the risk of postoperative infection in thoracic (RR 0.38; 95% CI: 0.18–0.78) and liver surgery (RR 0.39; 95% CI: 0.29–0.52). In patients undergoing esophageal (RR 0.89; 95% CI: 0.80–0.99) and gastrointestinal (RR 0.28; 95% CI: 0.23–0.34) surgery with a BMI greater than 25, omentoplasty is significantly associated with a reduced risk of overall complications compared to patients with normal BMI. No significant differences were found in pelvi-perineal surgery, except infection in patients whose BMI ranged from 25 kg/m^2^ to 29.9 kg/m^2^ (RR 1.25; 95% CI: 1.04–1.50) and anastomotic leakage in patients aged over 60 (RR 0.59; 95% CI: 0.39–0.91).

**Conclusion:**

Omentoplasty can effectively prevent postoperative infection. It is associated with a lower incidence of multiple postoperative complications in gastrointestinal and liver surgery.

## Introduction

Highlights

- First meta-analysis to provide a comprehensive summary of omentoplasty in various surgical procedures.
- The effect of BMI and age on postoperative outcomes varies in different surgeries.
- Omentoplasty provides no significant benefits in pelvi-perineal surgery.

Omentum is a type of tissue found within the abdominal cavity. It consists primarily of adipose tissue and blood vessels. Recent researches have revealed that omentum tissue is closely associated with immunity. For instance, neutrophils promote tumor metastasis to omentum tissue, whereas autologous omentum tissue transplantation reduced inflammation after kidney damage^1,2^. Omentoplasty is a surgical procedure in which a portion of the omentum is used to cover or fill a defect, improve arterial or portal circulation, absorb fluid, or increase lymphatic drainage^3^. It is widely used in various surgical procedures, including head trauma, chest wall infection, breast reconstruction, esophageal cancer resection, mediastinal infection, gastric cancer resection, sleeve gastrectomy, hepatic echinococcosis cyst resection, colorectal cancer resection, pelvi-perineal reconstruction, and so on.

Previous meta-analyses and systematic reviews of omentoplasty have provided valuable recommendations for clinical practice^3–9^. Nevertheless, certain controversial and unresolved issues remain in these studies. For instance, previous studies found no difference in the incidence of various clinical outcomes between omentoplasty and nonomentoplasty patients undergoing colorectal surgery^6,8^. On the contrary, recent research has shown that omentoplasty can reduce the incidence of anastomotic leaks after colorectal surgery^9^. Similar divergent opinions exist in esophageal surgery regarding the incidence of anastomotic leakage after omentoplasty following esophagectomy. Some studies indicated a positive impact^3–6,10^, while others indicated no significant difference^11^. Moreover, the effect of omentoplasty on postoperative infections varies depending on the specific surgical procedure. For instance, omentoplasty does not affect the incidence of the chest wall or mediastinal infection in cases involving omental flap reconstruction or pectoralis major muscle flap reconstruction^12^, but it does reduce the incidence of deep organ space infections in liver surgery among patients with hepatic echinococcosis^13^. There are also differing opinions regarding the clinical outcomes of complications such as postoperative recurrence rate, mortality, and so on, when omentoplasty is used^7^. Regarding the application of omentoplasty, opinions vary when analyzing the complications and clinical outcomes of various surgical procedures. By analyzing the existing studies on omentoplasty, we found that age and BMI are frequently neglected. These factors may have contributed to the inconsistency in recent clinical studies, as they are typically regarded as significant in determining surgical outcomes. In addition, due to the small sample size of previous analyses, there has yet to be a comprehensive examination of the advantages and disadvantages of incorporating omentoplasty into various surgical procedures. Consequently, the timing and scope of omentoplasty, despite being essential for guiding clinical applications, have yet to be specified.

To investigate the true role of omentoplasty in various surgical procedures and to determine if age and BMI have an impact on the different complications associated with omentoplasty, our analysis aimed to include additional relevant literature through a comprehensive literature search. In this meta-analysis, we considered esophageal, thoracic, gastrointestinal, liver, and pelvi-perineal surgical interventions. To provide a comprehensive summary of the optimal clinical application scenarios for omentoplasty, we analyzed the influence of omentoplasty on postoperative complications in these surgeries. This investigation may serve as a useful guide for future clinical procedures.

## Methods

This systematic review and meta-analysis was registered in the International Prospective Register of Systematic Reviews. The study followed the guideline statement provided by Preferred Reporting Items for Systematic Reviews and Meta-analyses (PRISMA, Supplemental Digital Content 1, http://links.lww.com/JS9/C61, Supplemental Digital Content 2, http://links.lww.com/JS9/C62) and A MeaSurement Tool to Assess systematic Reviews (AMSTAR 2, Supplemental Digital Content 3, http://links.lww.com/JS9/C63)^14,15^. Since this study incorporated secondary data from other investigations, informed permission, and ethical approval were waived.

### Search strategy and selection criteria

The literature search about omentoplasty was conducted with four databases, namely PubMed, Web of Science, the Cochrane Library, and Embase, on 14 July 2022. The search terms used for the retrieval included ‘Omentoplasty’, ‘Omentopexy’, ‘Epiploplasty’, ‘Omental Flap’, ‘Omentum Flap’, ‘Omental wrapping’, ‘Omentum plasty’, etc. Detailed retrieval strategies can be found in SDC, Table S1 in the Supplement (Supplemental Digital Content 4, http://links.lww.com/JS9/C64).

The included articles focused primarily on five major surgical interventions performed in conjunction with omentoplasty, including thoracic surgery, esophageal surgery, gastrointestinal surgery, pelvi-perineal surgery, and liver surgery. Among these, colorectal and anal operations were classified as pelvi-perineal operations. The inclusion and classification for these articles were primarily based on the clinical application direction and significance of omentoplasty.

Furthermore, non-English articles, publications before 1990, animal studies, studies without original data, case reports or case series, case–control studies, small sample studies (*n*<10), and studies in which omentoplasty was not considered as a single variable were excluded.

### Outcomes

The primary objective of this meta-analysis was to compare the overall incidence of postoperative complications between different types of surgical interventions with and without omentoplasty. Additionally, we conducted a comprehensive analysis of other outcomes such as postoperative infection, anastomotic leakage or fistula, death, recurrence, length of hospital stay, and so on, considering the unique circumstances of various surgeries. The relative risk (RR), which represents the ratio of the incidence of postoperative complications with omentoplasty to the incidence of complications without omentoplasty, was utilized in the analysis of postoperative complications. The mean difference was applied for the analysis of hospital stay, which represents the arithmetic mean of the absolute value of the deviation between the length of hospital stays and the mean. The difference in mean difference between patients undergoing omentoplasty and patients without omentoplasty was indicative of the effect of omentoplasty. Moreover, we specifically investigated the effects of age and BMI on the outcomes of different surgical interventions.

### Data collection and extraction

Two review authors initially screened the titles and abstracts of the retrieved articles, including studies from additional sources, to identify potentially relevant studies meeting the specified inclusion criteria. The full texts of these potentially eligible studies were then independently reviewed by the authors to assess their eligibility. The literature selection process is presented in an adjusted PRISMA flowchart. Data extraction was performed independently by two reviewers using a standardized predesigned data extraction form. These extracted data included study design, participant demographics, interventions, surgical details, baseline characteristics, postoperative outcomes, and other basic information about the studies retrieved. Discrepancies between the two authors were resolved by discussion with a third reviewer.

### Assessment of risk of bias in included studies

The assessment of the risk of bias in the included studies was conducted independently by two authors using the Cochrane risk of bias tool (Cochrane ROB tool2) for randomized controlled trials or the Newcastle–Ottawa Scale (NOS) for nonrandomized studies^16,17^. Disagreements between the two authors’ assessments were resolved through discussion with a third reviewer.

### Data synthesis and analysis

All of the included studies were divided into five categories based on the type of surgery performed, including gastrointestinal, liver, pelvi-perineal, esophageal, and thoracic surgery. Due to the diverse characteristics of each surgical procedure, the postoperative outcomes discussed in each category analysis varied. In gastrointestinal surgery, the incidence of overall complications, postoperative bleeding, infection, anastomotic leakage, fistula, delayed gastric emptying, mortality, and the mean difference in hospital stay were the primary variables investigated. The major variables studied in liver surgery were the incidence of overall complications, infection, anastomotic leakage, fistula, recurrence, mortality, and the mean difference in hospital stay. In pelvi-perineal surgery, the main postoperative outcomes analyzed included the incidence of overall complications, postoperative bleeding, infection, wound dehiscence, anastomotic leakage, ileus, reoperation, and mortality. The major variables evaluated in esophageal surgery included the incidence of overall complications, infection, anastomotic leakage, and mortality. In thoracic surgery, the main analyzed outcomes included the incidence of overall complications, infection, reoperation, mortality, and the mean difference in hospital stay. The intervention effects of each study were analyzed using Review Manager (RevMan 5.4; Cochrane Collaboration). For dichotomous data, pooled RR and 95% CIs were calculated using random or fixed-effects models, while mean differences were employed for continuous data. Assessment of statistical heterogeneity using *Q*-test and *I*^2^ statistic. The random-effects model was applied when *I*^2^ >50%, and the fixed-effects model was utilized when *I*^2^ ≤50%. To mitigate bias associated with different BMIs or ages and to explore the impact of diverse BMIs and ages on omentoplasty outcomes, subgroup analyses were performed. Patients were categorized into three BMI subgroups: 18.5–24.9 kg/m², 25–29.9 kg/m², and ≥30 kg/m². Similarly, based on age, patients were divided into two subgroups: <60 years old and ≥60 years old. Publication bias was assessed visually using funnel plots and statistically using the Egger’s test with Review Manager and Stata with 10 or more included studies. Publication bias was deemed statistically significant when the *P*-value was 0.05 or below. Meta-regression analyses were conducted with Stata 16 to detect possible influential factors. Sensitivity analyses were performed by omitting each study iteratively for each postoperative outcome with Stata 16.

## Results

### Literature search and selection

**Figure 1:**
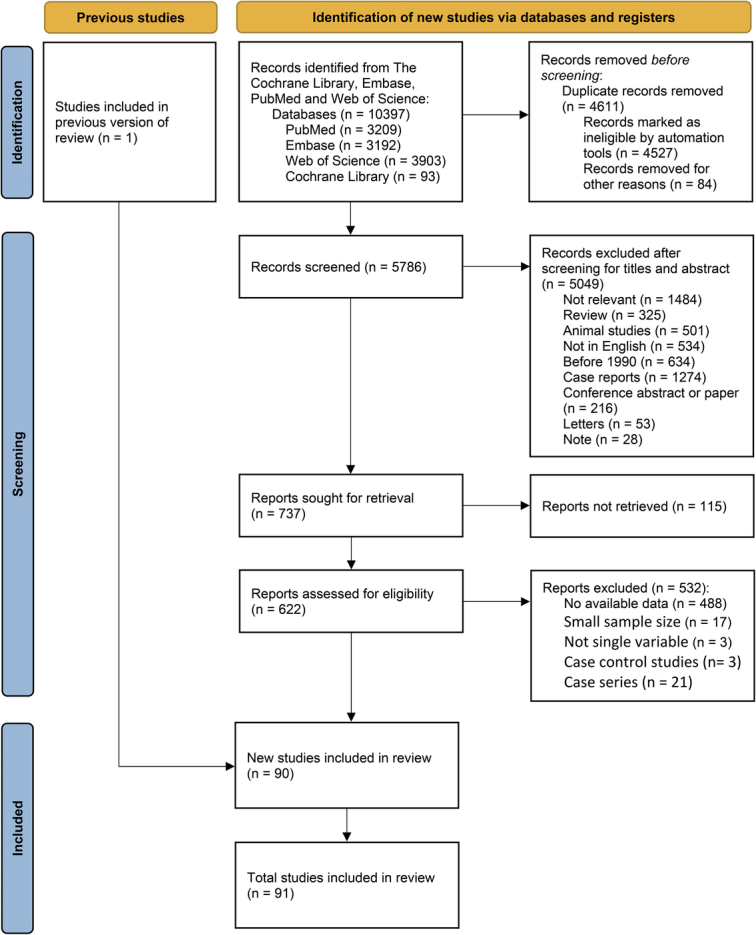
PRISMA (preferred reporting items for systematic reviews and meta-analyses) 2020 flow diagram for systematic reviews shows the procedure of literature search and processing of this study. Initially, reviewers identified relevant records using the strategy described in the methods section and manually and automatically excluded duplicates. Secondly, reviewers screened records for their titles and abstracts before excluding those that did not pertain to the study. Finally, reviewers evaluated the eligibility of the retrieved reports by examining the full text, and 91 of the studies met the criteria. This review included a total of 92 studies, one of which was included in the previous review.

A total of 10 397 articles were retrieved from four databases (Fig. 1). After removing duplicates, 5786 literatures remained. After reviewing titles and abstracts, 5049 articles were excluded, including reviews, animal studies, non-English-language studies, articles published before 1990, case reports, conference abstracts or papers, letters, notes, and irrelevant studies. Upon a thorough review of the full text, 91 studies were finally included.

### Study characteristics

The basic characteristics of the studies included in this paper are depicted in Table 1. The studies were divided into five categories. Thoracic surgeries mainly included chest wall infection, mediastinitis, chest wall reconstruction, breast reconstruction, etc. Esophageal surgeries mainly included esophagogastrostomy after esophageal cancer surgery, esophageal reconstruction, etc. Gastrointestinal surgeries included operations of the upper digestive tract except for the esophagus, such as peptic ulcer perforation repair, sleeve gastrectomy, pancreaticoduodenectomy, etc. Pelvi-perineal surgeries mainly included pelvi-perineal reconstructions after abdominal perineal resection and reconstructions after colorectal resection. Liver surgeries mainly included radical operation of hepatic echinococcosis and hepatectomy.

**Table 1:** Summary of characteristics of included studies^a^.

			Patients					Patients	
Surgery	Study (Author)	Year	OP	NOP	Surgery	Study (Author)	Year	OP	NOP
Esophageal	Bhat *et al*.^18^	2006	97	97	Pelvi-perineal	Agnifili *et al*.^19^	2004	62	64
	Dai *et al*.^20^	2011	128	127		Blok *et al*.^21^	2019	32	68
	Lu *et al*.^11^	2020	147	98		Blok *et al*.^22^	2019	106	148
	Sepesi *et al*.^23^	2012	215	392		Blok *et al*.^24^	2018	172	305
	Slaman *et al*.^25^	2022	290	39		Chaudhry *et al*.^26^	2019	288	274
	Ye *et al*.^27^	2016	121	87		Hultman *et al*.^28^	2010	29	41
	Zheng *et al*.^29^	2013	92	92		John and Buchman^30^	1991	38	36
	Zhou *et al*.^31^	2018	87	73		Lefevre *et al*.^32^	2009	52	43
thoracic	Aukema *et al*.^33^	2009	60	28		Meng *et al*.^34^	2021	17	47
	Barnea *et al*.^35^	2000	15	15		Merad *et al*.^36^	1998	341	364
	El-Sherpiny *et al*.^37^	2021	24	20		Miyamoto *et al*.^38^	2016	10	17
	Hashimoto *et al*.^39^	2014	10	30		Nagata *et al*.^40^	2020	28	14
	López-Monjardin *et al*.^41^	1998	12	21		Ngan *et al*.^42^	2008	46	51
	Marzouk *et al*.^43^	2021	33	364		Ozben *et al*.^44^	2016	65	403
	Milano *et al*.^45^	1999	21	38		Ozben *et al*.^46^	2018	86	2805
	Rouanet *et al*.^47^	1995	20	100		Poston *et al*.^48^	1991	28	25
	Soto *et al*.^49^	2022	47	58		Singh *et al*.^50^	2019	35	34
	Tewarie *et al*.^51^	2019	19	20		Tocchi *et al*.^52^	2000	53	59
	Zhou and Zhang^53^	2019	25	25		Welten *et al*.^54^	2019	173	2890
gastrointestinal	Abd Ellatif *et al*.^55^	2013	108	71	liver	Agaoglu *et al*.^56^	2003	16	28
	Abdallah *et al*.^57^	2020	54	52		Abdelraouf *et al*.^58^	2015	23	17
	Abosayed and Mostafa^59^	2022	45	46		Bhat *et al*.^60^	2020	30	27
	Afaneh *et al*.^61^	2015	30	30		Borham MM^62^	2014	32	28
	AlHaddad *et al*.^63^	2021	70	70		Dziri *et al*.^64^	1999	58	57
	Deng *et al*.^65^	2022	86	89		Erdem *et al*.^66^	1998	21	28
	Fouad *et al*.^67^	2022	419	406		Erdener *et al*.^56^	1992	12	27
	Husain *et al*.^68^	2011	90	86		Gourgiotis *et al*.^69^	2007	72	22
	Kim MG^70^	2015	56	14		Hamamci *et al*.^71^	2005	16	23
	Lale *et al*.^72^	2020	1574	962		Igami *et al*.^73^	2011	20	20
	Li *et al*.^74^	2022	69	69		Kayaalp *et al*.^75^	2002	16	51
	Lin *et al*.^76^	2017	91	27		Kouraklis *et al*.^77^	2005	33	12
	Lo *et al*.^78^	2011	47	26		Manterola *et al*.^79^	2013	48	40
	Matsuda *et al*.^80^	2012	157	72		Muftuoglu *et al*.^81^	2005	156	32
	Naga *et al*.^82^	2020	50	50		Nanashima *et al*.^83^	2012	14	65
	Nasiri *et al*.^84^	2017	62	62		Okano *et al*.^85^	2013	25	25
	Negm *et al*.^86^	2022	50	50		Ozacmak *et al*.^87^	2000	35	73
	Nosrati *et al*.^88^	2021	100	101		Panaro *et al*.^89^	2014	62	50
	Ölmez *et al*.^90^	2019	243	46		Paquet *et al*.^91^	2000	87	80
	Pan *et al*.^92^	2020	49	30		Pechlivanides *et al*.^93^	1991	95	57
	Pilone *et al*.^94^	2019	96	90		Reza Mousavi *et al*.^95^	2005	35	30
	Rosso *et al*.^96^	2012	33	28		Takatsuki *et al*.^97^	2021	50	13
	Sabry and Qassem^98^	2018	1000	1000		Tsaroucha *et al*.^99^	2005	25	32
	Shah *et al*.^100^	2015	101	46		Wani *et al*.^101^	2013	22	28
	Smith D^102^	2018	77	17		Xu S^103^	2020	25	24
	Tangtawee *et al*.^104^	2021	34	34		Zaouche *et al*.^105^	2001	28	49
	Tani *et al*.^106^	2012	699	1679					

a This review included 25 324 patients from 92 studies, among which 9701 patients underwent omentoplasty. In the esophageal surgery group, a total of 1177 patients underwent omentoplasty while 1005 did not. In the thoracic surgery group, a total of 286 patients underwent omentoplasty while 719 did not. In the gastrointestinal surgery group, a total of 5490 patients underwent omentoplasty while 5253 did not. In the liver surgery group, a total of 1056 patients underwent omentoplasty while 983 did not. In the pelvi-perineal surgery group, a total of 1692 patients underwent omentoplasty while 7708 did not.

NOP, nonomentoplasty; OP, omentoplasty.

### Assessment of risk of bias

Table S2 in the Supplemental Digital Content 5, http://links.lww.com/JS9/C65 provided a detailed quality evaluation. The quality evaluation of the literatures we included was basically above the medium level to ensure the quality of the research. The mean NOS score for 70 cohort studies was 6.9 (SD 0.9; range 5–9), where the main risk of bias focused on the representativeness of the exposed cohorts and the comparability of the cohorts. Four of 18 randomized controlled trials had high risks of bias: two in allocation concealment and the other two in blinding of participants and personnel. The mean MINORS score for all three nonrandomized clinical trials was 17.7 (SD 1.7; range 16–20).

### Gastrointestinal surgery

Twenty-seven studies on gastrointestinal surgery were identified^55,57,59,61,63,65,67,68,70,72,74,76,78,80,82,84,86,88,90,92,94,96,98,100,102,104,106^, including repair of perforated peptic ulcers, laparoscopic sleeve gastrectomy, and pancreaticoduodenectomy. A total of 10 743 participants were included, of which 5490 underwent omentoplasty and 5253 did not.

**Figure 2:**
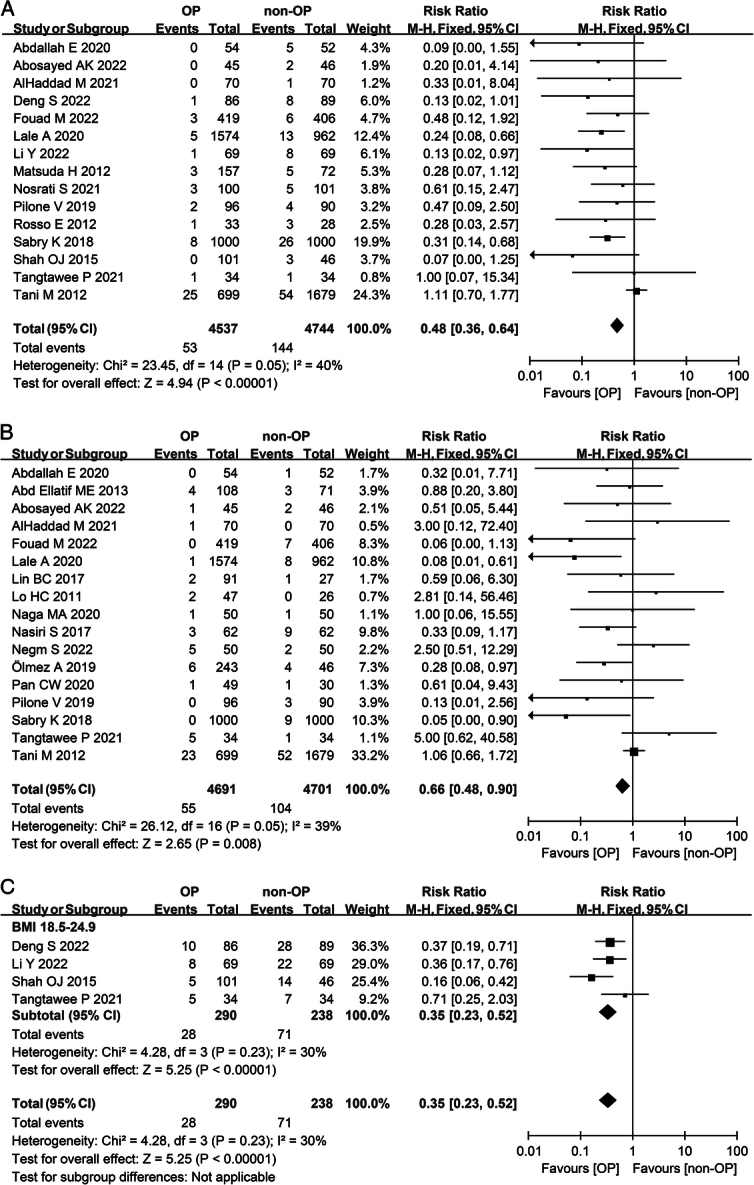
Meta-analysis of postoperative results of gastrointestinal surgery with or without omentoplasty. (A) Forest plot illustrating the incidence of postoperative bleeding after gastrointestinal surgery in a fixed-effects model. (B) Forest plot illustrating the incidence of anastomotic leak after gastrointestinal surgery in a fixed-effects model. (C) Forest plot illustrating the subgroup analysis of BMI on the incidence of fistula after gastrointestinal surgery in a fixed-effects model. Patients whose BMI was over 25 were not included in this subgroup analysis due to a lack of data. OP, omentoplasty; non-OP, nonomentoplasty.

In comparison to the nonomentoplasty group, the overall complication rate in the omentoplasty group was significantly lower (RR 0.53; 95% CI: 0.39–0.72; *I*^2^=85%) (Table 2). Patients who received omentoplasty had a significantly reduced incidence of postoperative bleeding (RR 0.48; 95% CI: 0.36–0.64; *I*^2^=40%) (Fig. 2A) and anastomotic leakage (RR 0.66; 95% CI: 0.48–0.90; *I*^2^=39%) (Fig. 2B) than those who did not. No significant difference between the two groups in the incidence of infection (RR 0.78; 95% CI: 0.56–1.08; *I*^2^=57%) (Table 2), fistula (RR 0.58; 95% CI: 0.34–1.00; *I*^2^=84%) (Table 2), delayed gastric emptying (RR 0.71; 95% CI: 0.36–1.39; *I*^2^=55%) (Table 2) or mortality (RR 0.69; 95% CI: 0.44–1.09; *I*^2^=3%) (Table 2) was evident. Patients with omentoplasty had fewer hospital days than those without omentoplasty (MD −1.05; 95% CI: −1.65 to −0.45; *I*^2^= 96%) (Table 2).

**Table 2:** Results summary^a^.

Surgery	Overall complications (RR;95% CI)P^b^	Infection (RR;95% CI)P	Anastomotic leakage (RR;95% CI)P	Mortality (RR;95% CI)P	Reoperation (RR;95% CI)P	Postoperative bleeding (RR;95% CI)P	Fistula (RR;95% CI)P	Delayed gastric emptying (RR;95% CI)P	Hospital stay (MD^c^;95% CI)P	Recurrence (RR;95% CI)P	Wound dehiscence (RR;95% CI)P	Ileus (RR;95% CI)P
Gastrointestinal surgery												
Total	↓^d^ (0.53;0.39–0.72)^***^	— ^e^(0.78;0.56–1.08)0.13	↓(0.66;0.48–0.90)^**^	—(0.69;0.44–1.09)0.11	NA^f^	↓(0.48;0.36–0.64)^***^	—(0.58;0.34–1.00)^**^	—(0.71;0.36–1.39)0.32	↓(-1.05;-1.65~-0.45)^***^	NA	NA	NA
BMI 18.5–24.9	↓(0.41;0.33-0.51)^g^ ^***^	—(0.68;0.45-1.03)0.07	NA	—(0.34;0.10-1.14)0.08	NA	↓(0.20;0.07-0.57)^**^	↓(0.35;0.23–0.52)^***^	NA	NA	NA	NA	NA
BMI≥30	↓(0.28;0.23–0.34)^g^ ^***^	NA	↓(0.17;0.08-0.40)^***^	NA	NA	↓(0.32;0.20-0.51)^***^	NA	NA	↓ (−0.29;-0.35~-0.24)^***^	NA	NA	NA
Age<60	↓(0.44;0.38-0.50)^h^ ^***^	↓(0.69;0.53-0.89)^**^	↓(0.38;0.24-0.60)^***^	—(0.76;0.33-1.77)0.52	NA	↓(0.32;0.20-0.51)^***^	NA	NA	↓ (−0.29;-0.35~-0.24)^***^	NA	NA	NA
Age≥60	↓(0.44;0.33-0.58)^h^ ^***^	—(0.54;0.25-1.14)0.10	NA	—(0.34;0.09-1.27)0.11	NA	↓(0.14;0.04-0.52)^**^	↓(0.35;0.21-0.59)^***^	↓(0.37;0.17-0.82)^*^	↓ (−1.17;-2.27~-0.07)^*^	NA	NA	NA
Liver surgery												
Total	↓(0.54;0.39–0.74)^***^	↓(0.39;0.29–0.52)^***^	—(0.94;0.57–1.56)0.81	—(0.60;0.30–1.17)0.13	NA	NA	↓(0.43;0.18–0.99)0.05	NA	↓ (−5.18;-8.53~-1.83)^**^	↓(0.19;0.09–0.41)^***^	NA	NA
Age<60	↓(0.63;0.50-0.80)^***^	↓(0.46;0.27-0.80)^**^	—(0.98;0.51-1.89)0.95	—(0.61;0.27-1.39)0.24	NA	NA	—(0.63;0.28-1.41)0.27	NA	↓ (−4.40;-5.26~-3.54)^***^	—(0.18;0.02-1.46)0.11	NA	NA
Age≥60	NA	—(1.00;0.36-2.79)1.00	—(1.00;0.38-2.62)1.00	NA	NA	NA	NA	NA	— (−0.02;-0.84~0.79)0.96	NA	NA	NA
Pelvi-perineal surgery												
Total	—(0.96;0.76–1.20)0.71	—(1.11;0.87–1.41)0.42	—(0.68;0.38–1.19)0.18	—(0.85;0.53–1.39)0.52	—(1.05;0.80–1.38)0.74)	—(0.83;0.42–1.64)0.59	NA	NA	NA	NA	—(0.63;0.38–1.05)0.08	—(0.93;0.76–1.14)0.49
BMI18.5–24.9	↑^i^ (1.18;1.04–1.35)^*^	NA	NA	NA	NA	NA	NA	NA	NA	NA	NA	NA
BMI25-29.9	—(1.29;0.98-1.69)0.07	↑(1.25;1.04–1.50)^*^	NA	NA	NA	NA	NA	NA	NA	NA	NA	NA
Age<60	—(1.00;0.81-1.23)1.00	—(0.87;0.57–1.35)0.54	—(1.19;0.63-2.27)0.59	—(0.62;0.19-1.98)0.42	—(1.23;0.69-2.20)0.47	NA	NA	NA	NA	NA	NA	—(0.67;0.37-1.21)0.18
Age≥60	—(1.03;0.91-1.16)0.68	—(1.09;0.88-1.35)0.41	↓(0.59;0.39–0.91)^*^	—(1.01;0.58–1.75)0.98	—(1.01;0.73-1.38)0.97	—(0.88;0.43-1.82)0.74	NA	NA	NA	NA	↓(0.66;0.47-0.92)^*^	—(0.97;0.78-1.21)0.81
Esophageal surgery												
Total	↓(0.79;0.72–0.88)^***^	—(0.77;0.58–1.01)^*^	↓(0.37;0.19–0.72)^**^	—(0.73;0.32–1.69)0.46	NA	NA	NA	NA	NA	NA	NA	NA
BMI25-29.9	↓(0.89;0.80–0.99)^*^	NA	NA	NA	NA	NA	NA	NA	NA	NA	NA	NA
Age≥60	↓(0.80;0.71-0.89)^***^	—(0.79;0.59–1.04)0.09	↓(0.52;0.28-0.97)^*^	↓(0.52;0.28-0.97)^*^	NA	NA	NA	NA	NA	NA	NA	NA
Thoracic surgery												
Total	—(1.22;0.84–1.75)0.29	↓(0.38;0.18–0.78)^**^	NA	—(1.06;0.59–1.92)0.84	—(1.02;0.64–1.63)0.92	NA	NA	NA	↑(10.96;1.07~20.84)^*^	NA	NA	NA
BMI≥30	NA	NA	NA	—(1.29;0.60-2.78)0.52	NA	NA	NA	NA	NA	NA	NA	NA
Age<60	—(1.15;0.93-1.43)0.19	NA	NA	NA	NA	NA	NA	NA	NA	NA	NA	NA
Age≥60	↑(1.20;1.01–1.44)^*^	↓(0.34;0.12-0.93)^*^	NA	—(1.13;0.62-2.08)0.69	—(1.32;0.64-2.74)0.45	NA	NA	NA	NA	NA	NA	NA

a A summary of the results of postoperative complications and BMI, age subgroup analyses for the five surgical procedures with or without omentoplasty is presented in this table.

b RR, relative risk.

c MD, mean difference.

d The result favored omentoplasty.

e No significant difference between with or without omentoplasty.

f NA, not applicable.

g Significant difference between two BMI subgroups.

h Significant difference between two age subgroups.

i The result favored nonomentoplasty.

* *P*<0.05.

** *P*<0.01.

*** *P*<0.001.

### Liver surgery

Twenty-six studies on liver surgery were included^56,58,60,62,64,66,69,71,73,75,77,79,81,83,85,87,89,91,93,95,97,99,101,103,105,107^, with the majority of surgical interventions relating to hepatic hydatid cysts. This study included 1994 individuals, 1056 of whom had omentoplasty and 938 did not.

**Figure 3:**
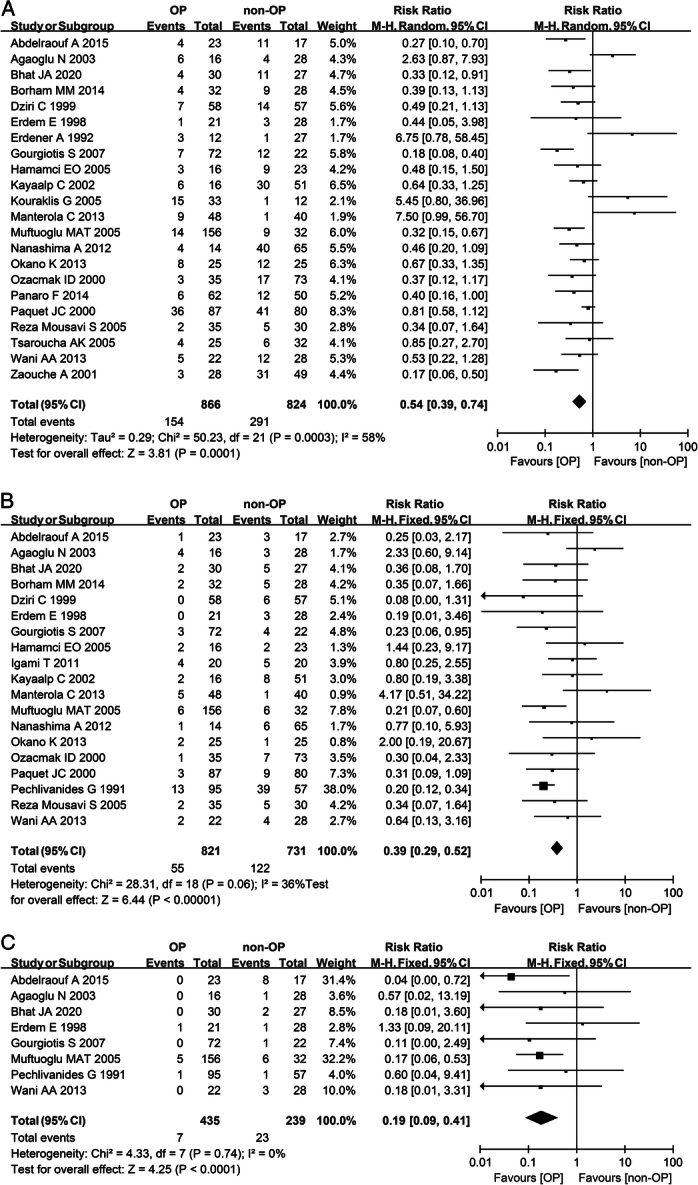
Meta-analysis of postoperative results of liver surgery with or without omentoplasty. (A) Forest plot illustrating the incidence of infection after liver surgery in a fixed-effects model. (B) Forest plot illustrating the incidence of recurrence after liver surgery in a fixed-effects model. (C) Forest plot illustrating the subgroup analysis of age on mortality after liver surgery in a fixed-effects model. Patients aged over 60 were not included in this subgroup analysis due to a lack of data OP, omentoplasty; non-OP, nonomentoplasty.

With a relatively high heterogeneity, the overall complication rate (RR 0.54; 95% CI: 0.39–0.74; *I*^2^=58%) (Fig. 3A) and fistula rate (RR 0.43; 95% CI: 0.18–0.99; *I*^2^=60%) (Table 2) of patients in the omentoplasty group were nearly half of those in the nonomentoplasty group, showing significant differences. Patients who underwent omentoplasty had a greatly lower incidence of infection (RR 0.39; 95% CI: 0.29–0.52; *I*^2^=36%) (Fig. 3B) and recurrence (RR 0.19; 95% CI: 0.09–0.41; *I*^2^=0%) (Fig. 3C) than those who did not. Patients with omentoplasty also had a reduction in hospital days (MD −5.18; 95% CI: −8.53 to −1.83; *I*^2^=97%) (Table 2). There was no significant difference between the two groups in the incidence of anastomotic leakage (RR 0.94; 95% CI: 0.57–1.56; *I*^2^=0%) (Table 2) or mortality (RR 0.60; 95% CI: 0.30–1.17; *I*^2^=19%) (Table 2). A subgroup analysis was performed to examine the outcomes of hepatectomy and radical operation of hepatic echinococcosis. It showed that the use of omentoplasty for these surgeries reduced the overall postoperative complication rate and the length of hospital stay; nevertheless, it had no effect on the mortality rate or the incidence of anastomotic leak, which did not differ significantly from the above results.

### Pelvi-perineal surgery

Nineteen studies on pelvi-perineal surgery were identified^19,21,22,24,26,28,30,32,34,36,38,40,42,44,46,48,50,52,54^, including 13 studies about reconstructions after abdominal perineal resection, five studies about resection of colorectal cancer and one about vesicovaginal fistula repairs. A total of 9349 people were investigated, with 1661 patients underwent omentoplasty and 7688 did not.

There was no significant difference found between the omentoplasty group and the nonomentoplasty group in overall complication rate (RR 0.96; 95% CI: 0.76–1.20; *I*^2^=82%) (Table 2). No significant difference between the two groups was found in the incidence of postoperative bleeding (RR 0.83; 95% CI: 0.42–1.64; *I*^2^=0%) (Table 2), infection (RR 1.11; 95% CI: 0.87–1.41; *I*^2^=55%) (Table 2), wound dehiscence (RR 0.63; 95% CI: 0.38–1.05; *I*^2^=52%) (Table 2), anastomotic leakage (RR 0.68; 95% CI: 0.38–1.19; *I*^2^=51%) (Table 2), ileus (RR 0.93; 95% CI: 0.76–1.14; *I*^2^=18%) (Table 2), reoperation (RR 1.05; 95% CI: 0.80–1.38; *I*^2^=37%) (Table 2) and mortality (RR 0.85; 95% CI: 0.53–1.39; *I*^2^=0%) (Table 2). We analyzed the outcomes of reconstructions after abdominal perineal resection and resection of colorectal cancer with or without the use of omentoplasty. The results were consistent with our previous pooled analysis that omentoplasty did not improve the occurrence of postoperative complications in patients.

### Esophageal surgery

Eight studies on esophageal surgery were included^11,18,20,23,25,27,29,31^, most of which were related to esophageal cancer. The average age of the patients enrolled was over 60 years. A total of 2182 participants were involved, with 1177 in the omentoplasty group and 1005 in the nonomentoplasty group.

**Figure 4:**
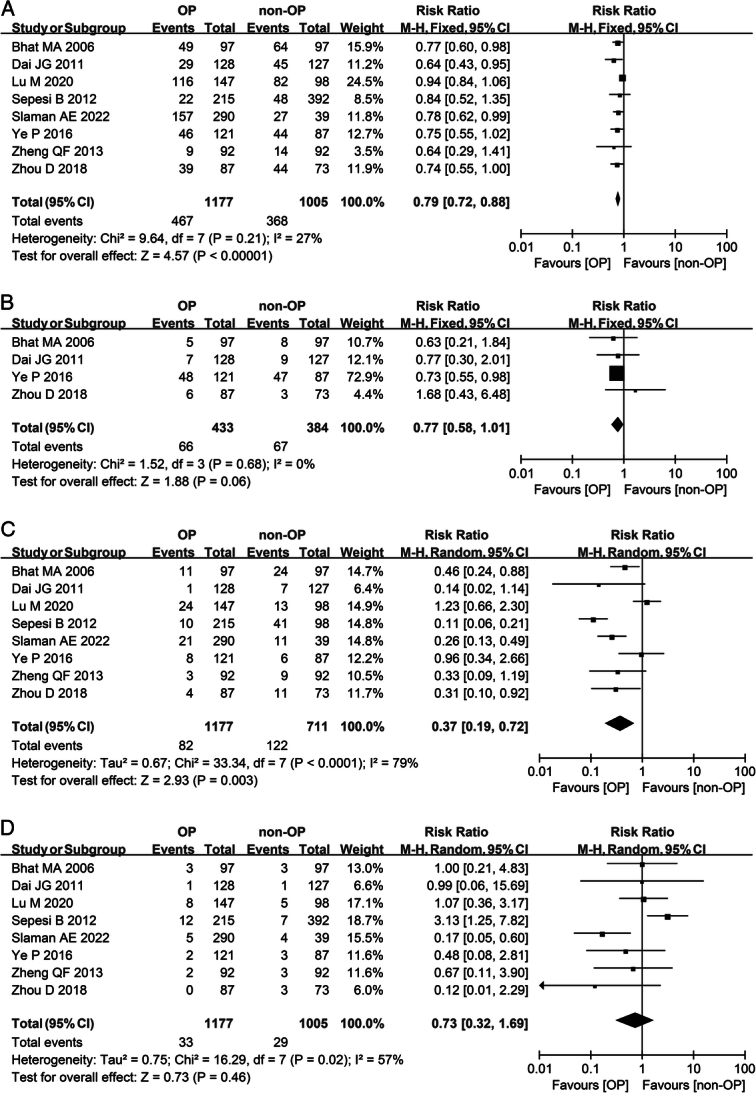
Meta-analysis of postoperative results of esophageal surgery with or without omentoplasty. (A) Forest plot illustrating the incidence of overall complications after esophageal surgery in a fixed-effects model. (B) Forest plot illustrating the incidence of infection after esophageal surgery in a fixed-effects model. (C) Forest plot illustrating the incidence of anastomotic leak after esophageal surgery in a random-effects model. (D) Forest plot illustrating the mortality after esophageal surgery in a random-effects model. OP, omentoplasty; non-OP, nonomentoplasty.

Compared with the control group, the omentoplasty treatment group showed a decreased risk of overall complications (RR 0.79; 95% CI: 0.72–0.88; *I*^2^=27%) (Fig. 4A). No significant difference was found in the incidence of postoperative infection between the two groups (RR 0.77; 95% CI: 0.58–1.01; *I*^2^=0%) (Fig. 4B). The omentoplasty group exhibited a lower incidence of postoperative anastomotic leakage compared to the nonomentoplasty group (RR 0.37; 95% CI: 0.19–0.72; *I*^2^=79%) (Fig. 4C). Regarding the incidence of postoperative mortality, no significant difference was observed between the two groups (RR 0.73; 95% CI: 0.32–1.69; *I*^2^=57%), but the results showed considerable heterogeneity (Fig. 4D).

### Thoracic surgery

Eleven studies on thoracic surgery were identified^33,35,37,39,41,43,45,47,49,51,53^, including seven sternal wound reconstructions and four breast reconstructions. A total of 1005 people were included, 286 of whom received omentoplasty and 719 of whom did not.

**Figure 5:**
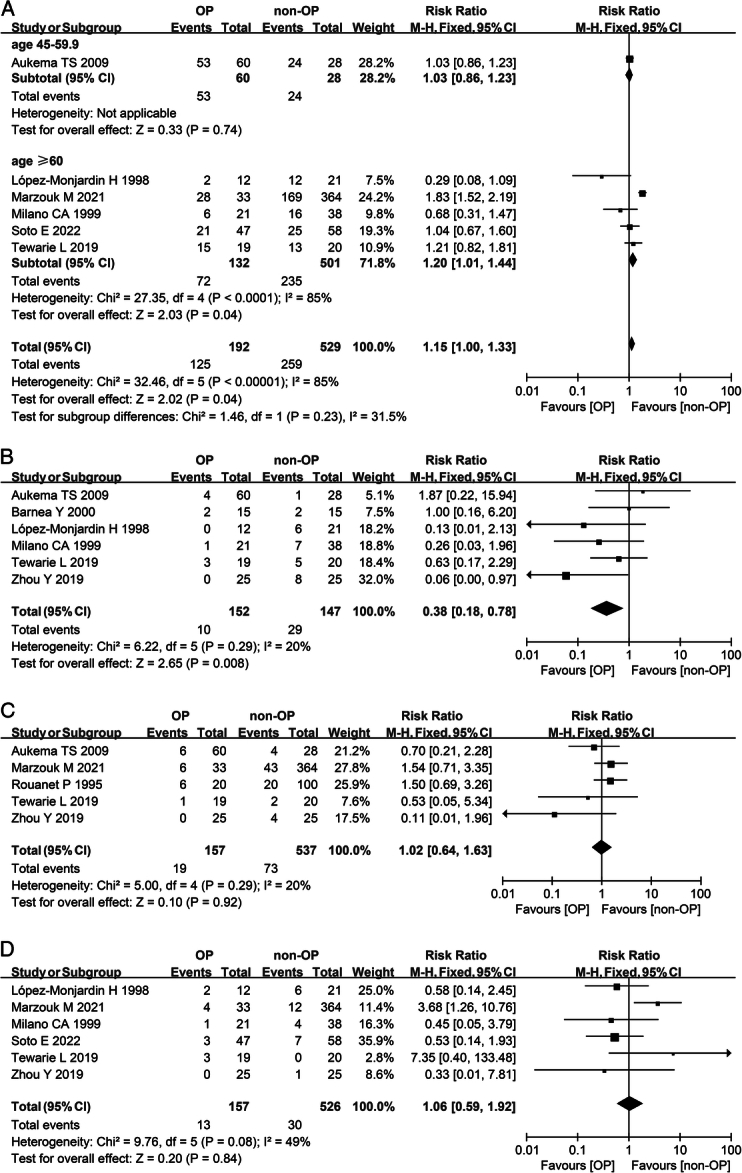
Meta-analysis of postoperative results of thoracic surgery with or without omentoplasty. (A) Forest plot illustrating subgroup analysis of age on overall complications after thoracic surgery in a fixed-effects model. Patients aged from 18 to 44.9 were not included in this subgroup analysis due to a lack of data. (B) Forest plot illustrating the incidence of infection after thoracic surgery in a fixed-effects model. (C) Forest plot illustrating the incidence of reoperation after thoracic surgery in a fixed-effects model. (D) Forest plot showing the mortality after thoracic surgery in a fixed-effects model. OP, omentoplasty; non-OP, nonomentoplasty.

No significant difference was evident in the overall complication rate between the omentoplasty group and the nonomentoplasty group (RR 1.22; 95% CI: 0.84–1.75; *I*^2^=87%) (Table 2). Compared to the control group, the incidence of infection was significantly lower in the omentoplasty group. (RR 0.38; 95% CI: 0.18–0.78; *I*^2^=20%) (Fig. 5B). There was no difference between the two groups in the incidence of reoperation (RR 1.02; 95% CI: 0.64–1.63; *I*^2^=20%) (Fig. 5C) or mortality (RR 1.06; 95% CI: 0.59–1.92; *I*^2^=49%) (Fig. 5D). The hospital days of patients in the omentoplasty group decreased compared to the control group (MD 10.96; 95% CI: 1.07–20.84; *I*^2^=94%) (Table 2). A meta-analysis was conducted on both of these procedures. The results did not change significantly from the previous pooled study and demonstrated that the use of omentoplasty had minimal effect on the occurrence of postoperative problems in patients, regardless of whether they were for breast reconstructions or sternal wound reconstructions.

### Subgroup analysis of BMI and age

**Figure 6:**
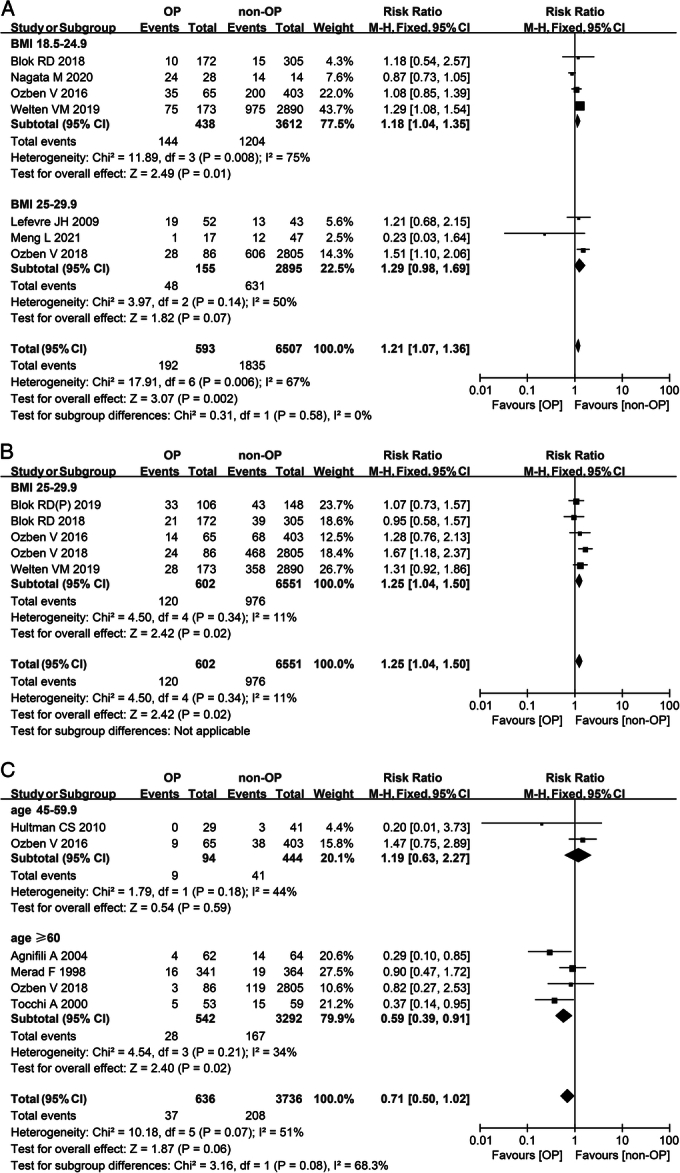
Meta-analysis of postoperative results of pelvi-perineal surgery with or without omentoplasty. (A) Forest plot illustrating the subgroup analysis of BMI on the incidence of overall complication after pelvi-perineal surgery in a fixed-effects model. Patients whose BMI was over 30 were not included in this subgroup analysis due to a lack of data. (B) Forest plot illustrating the subgroup analysis of BMI on the incidence of infection after pelvi-perineal surgery in a fixed-effects model. Patients whose BMI ranged from 18.5 to 24.9 or over 30 kg/m^2^ were not included in this subgroup analysis due to a lack of data. (C) Forest plot illustrating the subgroup analysis of age on the incidence of anastomotic leak after pelvi-perineal surgery in a fixed-effects model. Patients aged from 18 to 44.9 were not included in this subgroup analysis due to a lack of data. OP, omentoplasty; non-OP, nonomentoplasty.

For BMI subgroup analysis, in patients undergoing gastrointestinal surgery with a BMI ranged from 18.5 kg/m^2^ to 24.9 kg/m^2^, the incidence of the fistula was significantly lower in the omentoplasty group than in the nonomentoplasty group (RR 0.35; 95% CI: 0.23–0.52; *I*^2^=30%) (Fig. 2C). Interestingly, in patients with a BMI ranged from 18.5 kg/m^2^ to 24.9 kg/m^2^, who underwent pelvi-perineal surgery, the overall complication rate in the omentoplasty group was significantly higher than the nonomentoplasty group, despite the highly heterogenous results (RR 1.18; 95% CI: 1.04–1.35; *I*^2^=75%) (Fig. 6A). In patients whose BMI ranged from 25 kg/m^2^ to 29.9 kg/m^2^, the incidence of infection of patients who underwent omentoplasty was significantly higher than those who did not (RR 1.25; 95% CI: 1.04–1.50; *I*^2^=11%) (Fig. 6B).

For age subgroup analysis, in patients aged 60 or older who underwent thoracic surgery, the overall complication rate was significantly lower among those who did not use omentoplasty than among those who did, despite a high degree of heterogeneity (RR 1.20; 95% CI: 1.01–1.44; *I*^2^=85%) (Fig. 5A). In addition, in patients undergoing pelvi-perineal surgery who aged over 60, the incidence of anastomotic leakage was significantly lower in the omentoplasty group than in the nonomentoplasty group (RR 0.59; 95% CI: 0.39–0.91; *I*^2^=34%) (Fig. 6C).

### Analyses of publication bias, meta-regression, and sensitivity analyses

We used RevMan’s funnel plot and Stata’s Egger’s test to quantitatively assess publication bias for comparisons of omentoplasty or nonomentoplasty greater than 10 articles. The results of Stata’s Egger’s test were shown in SDC, Table S3 (Supplemental Digital Content 6, http://links.lww.com/JS9/C66) and funnel plots were displayed in SDC, Figure S1 (Supplemental Digital Content 7, http://links.lww.com/JS9/C67). No publication bias (*P*>0.05) (SDC, Table S3, Supplemental Digital Content 6, http://links.lww.com/JS9/C66) was observed in most articles we included, such as pelvic perineal surgery, and chest surgery. However, for gastrointestinal surgery, publication bias was seen when we analyzed postoperative bleeding (Egger’s test *P*=0.005) (SDC, Table S3, Supplemental Digital Content 6, http://links.lww.com/JS9/C66). In addition, the incidence of infection in patients with liver surgery had publication bias (Egger’s test *P*=0.045) (SDC, Table S3, Supplemental Digital Content 6, http://links.lww.com/JS9/C66).

To explain extreme heterogeneity, meta-regression analyses were done on hospital stay, revealing that none of the variables tested (study kind, geography, and study time) had a statistically significant influence (SDC, Table S4, Supplemental Digital Content 8, http://links.lww.com/JS9/C68).

In sensitivity analyses, a certain number of pooled estimates for various postoperative outcomes changed significantly after omitting a study (SDC, Table S5, Supplemental Digital Content 9, http://links.lww.com/JS9/C69). Notably, the results suggesting that omentoplasty brought no benefit accounted for the majority of unstable pooled estimates, which indicated significant differences in favor of omentoplasty after the exclusion of a particular study. (SDC, Figure S2, Supplemental Digital Content 10, http://links.lww.com/JS9/C70).

## Discussion

Although the impact of omentoplasty on surgical procedures has been extensively studied^3–8,13^, there is a lack of comprehensive studies that summarize all procedures and conduct subgroup analyses based on age and BMI. Both factors have significant implications for various postoperative outcomes^108–112^, necessitating further subgroup analysis to inform clinical decision-making. This is the first investigation into the current utilization of omentoplasty across a spectrum of procedures. Our findings support the widespread application of omentoplasty in esophageal, gastrointestinal, and liver surgeries, as it improves overall postoperative outcomes and decreases individual complications. However, a specific analysis of the type of complications, age, and BMI, is necessary for making decisions in thoracic and pelvi-perineal surgeries. According to our findings, patients undergoing surgery should take both age and BMI into careful consideration.

This study found that except for thoracic and pelvi-perineal surgery, the use of omentoplasty significantly reduces the incidence of overall complications for the other three procedures. Interestingly, in esophageal and gastrointestinal surgery, omentoplasty is associated with a significantly lower risk of overall complications in overweight and obese patients with a BMI greater than 25 kg/m^2^ compared to patients with a normal BMI. Although visceral adipose tissue (VAT) inflammation is significantly increased in the obese state, which is linked to hyperinsulinemia, metabolic syndrome, vascular risk, and cardiovascular events^113–115^, studies have reported that higher BMI in obese patients with rectal cancer does not harm long-term prognosis^116^, and obese patients may even exhibit a protective effect against sepsis mortality^117^. Therefore, one possible explanation is that the high levels of inflammatory factors in visceral fat in obese individuals allow for a prompt and protective response when the body experiences severe visceral damage. However, it should be emphasized that omentoplasty in overweight patients may increase the overall risk of pelvi-perineal surgery complications. In addition, it is essential to note that omentoplasty is not recommended for thoracic surgery patients over the age of 60 due to the increased risk of overall complications.

Postoperative infection is an important factor that affects the prognosis of clinical surgery for specific postoperative complications. Antibiotics are the main means to prevent postoperative infection, but it is necessary to carefully control the dose and duration^118^. In addition, omentoplasty has been suggested to have a role in the prevention of postoperative infection. In the current literature review, there are few studies examining the effect of omentoplasty on postoperative infection. Overall, we found that the use of omentoplasty in thoracic and liver surgeries significantly reduces the incidence of postoperative infections. This finding is consistent with the observation by Dziri *et al*.^13^ that omentoplasty can effectively reduce deep organ space surgical site infections in hepatic echinococcosis surgery. Notably, this benefit was diminished in patients older than 60 who had gastrointestinal and liver surgery. In addition, the use of omentoplasty in overweight patients undergoing pelvi-perineal surgery increases the risk of postoperative infection. Therefore, omentoplasty is not recommended for patients over 60 undergoing gastrointestinal and liver surgery, as well as overweight patients undergoing pelvic-perineal surgery, when the primary concern is a postoperative infection.

Except for thoracic surgery, omentoplasty reduces the incidence of postoperative anastomotic leakage complications in the other four operations to varying degrees. Regarding postoperative mortality, omentoplasty reduces mortality in patients over the age of 60 who have undergone esophageal surgery but does not affect other patients. In light of the two complications of anastomotic leakage and mortality, omentoplasty is recommended for all five surgical procedures. Furthermore, our analysis of procedure-specific postoperative complications revealed that omentoplasty decreased postoperative bleeding in gastrointestinal surgery but not in pelvi-perineal surgery. Moreover, we discovered that omentoplasty in liver surgery significantly reduced surgical recurrence. Lastly, in patients over 60 years old undergoing pelvi-perineal surgery, omentoplasty significantly reduced the degree of wound dehiscence. These findings suggest that depending on the type of surgery and the patient’s age, omentoplasty has variable but generally beneficial effects on these specific complications.

This study has several limitations to be addressed. Firstly, due to the comprehensive nature of the included literature, differences in follow-up durations, the availability of recorded postoperative outcomes, and variations in surgical techniques all contribute to inherent disparities among surgeries within the same category. The heterogeneity is particularly pronounced in surgical procedures, where different primary surgical types, including subgroup surgeries and diverse categories of surgeries, may impact the appropriateness of the omentoplasty. Therefore, the heterogeneity in surgical procedures remains a significant contributor to the high level of heterogeneity in the analysis results. To address this concern, subgroup, sensitivity, and meta-regression analyses were conducted to identify the potential causes of high heterogeneity. Additionally, efforts were made to refine the classification as much as possible to minimize differences among the surgical techniques included in this study.

Secondly, due to the limited number of available randomized controlled trials (RCT), it is difficult to conduct an exhaustive analysis using RCTs alone. Therefore, we have to combine RCTs with cohort studies for analysis, which may compromise the quality advantages and reduce the reliability of the analysis results. Thirdly, a small number of studies have reported BMI data, limiting the subgroup analysis results. However, we have made extensive efforts to collect all available BMI data to enhance the reliability of the subgroup analysis. Fourthly, sex-based subgroup analyses were not conducted. Since the primary outcome, such as the incidence of anastomotic leak, was pooled for all patients, we were unable to assess the difference in incidence between men and women separately.

In conclusion, this study involved a comprehensive analysis of clinical data that elucidated variations in the utilization of omentoplasty across various surgical procedures. This study aims to serve as a foundation for the implementation of omentoplasty in a variety of surgical settings, with a focus on the importance of considering overall patient characteristics, such as BMI and age. By taking these factors into account, informed decisions can be made regarding the application of omentoplasty, ultimately aiming to improve patient outcomes and well-being.

## Conclusions

Generally, omentoplasty effectively prevents postoperative infection. The application of omentoplasty in gastrointestinal and liver surgery significantly reduces the incidence of multiple postoperative complications. However, in pelvi-perineal surgery, omentoplasty provides no significant benefits and may even increase postoperative complications. After omentoplasty, thoracic surgery may be more prone to complications in patients over 60. In addition, BMI ≥25 kg/m^2^ increases the risk of postoperative infection in pelvi-perineal surgery with omentoplasty.

## Ethical approval

Since this study incorporated secondary data from other investigations, informed permission and ethical approval were waived.

## Consent

Since this study incorporated secondary data from other investigations, informed permission and ethical approval were waived.

## Sources of funding

This work was supported by the National Key R&D Program of China (2020YFA0803604), the National Natural Science Foundation of China, Key Program (82130024).

## Author contribution

Y.P., S.X., Y.D., L.X.: designed the research, performed literature review, analyzed results, made the figures and wrote the paper, they contributed equally to this work; T.D.: conceived and supervised the project, revised the manuscript, provided funding support; Y.W., Y.M., and W.L.: revised the manuscript. All authors read and approved the final manuscript. The corresponding author Tuo Deng attests that all listed authors meet authorship criteria and that no others meeting the criteria have been omitted.

## Conflicts of interest disclosure

All authors declare: supported by the National Key R&D Program of China, the National Natural Science Foundation of China, the Science and Technology Innovation Program of Hunan Province, and the Project of Innovation-Driven Plan of Central South University; no financial relationships with any organizations that might have an interest in the submitted work in the previous 3 years; no other relationships or activities that could appear to have influenced the submitted work.

## Research registration unique identifying number (UIN)

This systematic review and meta-analysis was registered in the International Prospective Register of Systematic Reviews (CRD42023349049).

## Guarantor

Tuo Deng.

## Data availability statement

The authors declare that all supporting data are available within the article (and its online supplementary files). Other Questions related to this article can be obtained by contacting the corresponding author at dengtuo@csu.edu.cn.

## Provenance and peer review

Not commissioned, externally peer-reviewed.

## Supplementary Material

**Figure:**