# Supplementary material for: The effect of omentoplasty in various surgical operations: systematic review and meta-analysis
Source: Int J Surg. 2024 Mar 4;110(6):3778–94. doi: 10.1097/JS9.0000000000001240 (PMC11175753; doi:10.1097/JS9.0000000000001240)
Supplement: Supplementary file 3 [file js9-110-3778-s004.pdf]

Table S1. Summary of the search strategy and results as of July 14th, 2022. The literatures involved in this review were identified independently from 4 databases on July 14th, 2022. A total of 10397 records were identified.

Datebases	Search query	Results
**PubMed**	(((((((Omentoplasty) OR (Omentopexy)) OR (Epiploplasty)) OR (Omental Flap)) OR (omentum flap)) OR (Omental wrapping)) OR (Omentum plasty)) OR (omental patch)	3209
**Embase**	'omentoplasty'/exp OR 'omentoplasty' OR 'omentopexy'/exp OR 'omentopexy' OR 'epiploplasty'/exp OR 'epiploplasty' OR 'omental flap'/exp OR 'omental flap' OR 'omentum flap'/exp OR 'omentum flap' OR 'omental wrapping'/exp OR 'omental wrapping' OR 'omentum plasty' OR 'omental patch'/exp OR 'omental patch'	3192
**Cochrane**	Omentoplasty or Omentopexy or Epiploplasty or Omental Flap or omentum flap or Omental wrapping or Omentum plasty or omental patch):ti,ab,kw (Word variations have been searched)" (Word variations have been searched)	93
**Web of Science**	(((((((TS=(Omentoplasty)) OR TS=(Omentopexy)) OR TS=(Epiploplasty)) OR TS=(Omental Flap)) OR TS=(Omentum flap)) OR TS=(Omental wrapping)) OR TS=(Omentum plasty)) OR TS=(omental patch)	3903
**Total**		10397
