# Supplementary material for: The effect of omentoplasty in various surgical operations: systematic review and meta-analysis
Source: Int J Surg. 2024 Mar 4;110(6):3778–94. doi: 10.1097/JS9.0000000000001240 (PMC11175753; doi:10.1097/JS9.0000000000001240)
Supplement: Supplementary file 4 [file js9-110-3778-s005.pdf]

Table S2. Quality evaluation of literatures included.

**A**		Cochrane ROB tool2						
		selection bias		performance bias	detection bias	attrition bias	reporting bias	other bias
Study	Research type	Random sequence generation	Allocation concealment	Blinding of participants and personnel	Blinding of outcome assessment	Incomplete outcome data	Selective reporting	Other bias sources
Afaneh C 2015	RCT	Unclear	Unclear	High	Low	Low	Unclear	Low
Negm S 2022	RCT	Low	Low	Unclear	Unclear	Low	Unclear	Unclear
Wani AA 2013	RCT	Low	Low	High	Unclear	Low	Unclear	Unclear
Abosayed AK 2022	RCT	Low	Low	Unclear	Unclear	Low	Unclear	Unclear
Pilone V 2019	RCT	Unclear	Unclear	Low	Unclear	Low	Unclear	Unclear
Zheng QF 2013	RCT	Low	Low	Unclear	Unclear	Low	Unclear	Unclear
Merad F 1998	RCT	Low	Low	Unclear	Unclear	Low	Unclear	Unclear
Dziri C 1999	RCT	Low	Low	Unclear	Unclear	Low	Unclear	Unclear
Paquet JC 2000	RCT	Low	Low	Unclear	Unclear	Low	Unclear	Unclear
Ngan HY 2008	RCT	Low	Low	Unclear	Low	Unclear	Unclear	Unclear
Tocchi A 2000	RCT	Low	High	Unclear	Unclear	Low	Unclear	Unclear
Singh V 2019	RCT	Low	Low	Unclear	Unclear	Low	Unclear	Unclear
Tangtawee P 2021	RCT	Low	Low	Unclear	Unclear	Low	Unclear	Unclear
Nasiri S 2017	RCT	Low	Low	Unclear	Low	Unclear	Unclear	Unclear
Agnifili A 2004	RCT	Low	High	Unclear	Unclear	Low	Unclear	Unclear
Bhat MA 2006	RCT	Low	Low	Unclear	Unclear	Low	Unclear	Unclear
Igami T 2011	RCT	Low	Low	Unclear	Unclear	Low	Unclear	Unclear
Dai JG 2011	RCT	Low	Low	Low	Low	Low	Unclear	Unclear

**B**			**MINORS(methodological index for non-randomized studies) scale**											
Study	Research type	Total score	A stated aim of the study	Inclusion of consecutive patients	Prospective collection of data	Endpoint appropriate to the study aim	Unbiased evaluation of endpoints	Follow-up period appropriate to the major endpoint	Loss to follow up not exceeding 5%	A control group having the gold standard intervention	Contemporary groups	Baseline equivalence of groups	Prospective calculation of the sample size	Statistical analyses adapted to the study design
Barnea Y 2000	clinical trial	16	2	2	2	2	1	0	2	2	2	1	0	0
El-Sherpiny WY 2021	clinical trial	20	2	2	2	2	1	1	2	2	2	2	0	2
Poston GJ 1991	clinical trial	17	2	2	1	2	1	1	2	2	0	1	0	1

C	Research type	Total score	NOS Cohort							
			Selection				Comparability	Outcome		
Study			Representativeness of the exposed cohort	Selection of the non exposed cohort	Demonstration that outcome of interest was not present at start of study	Ascertainment of exposure	Comparability of cohorts on the basis of the design or analysis	Assessment of outcome	Was follow-up long enough for outcomes to occur	Adequacy of follow up of cohorts
Lefevre JH 2009	retrospective cohort	8	※	※	※	※	※	※	※	※
Meng L 2021	retrospective cohort	7	0	※	※	※	※	※	※	※
Lu M 2020	retrospective cohort	7	※	※	※	※	※	※	0	※
Zhou D 2018	retrospective cohort	9	※	※	※	※	※※	※	※	※
Deng S 2022	retrospective cohort	7	※	※	※	※	※	※	0	※
Li Y 2022	retrospective cohort	7	※	※	※	※	※※	0	0	※
Fouad M 2022	prospective cohort	8	※	※	※	※	※	※	※	※
Nosrati S 2021	retrospective cohort	8	※	※	※	※	※	※	※	※
Rouanet P 1995	retrospective cohort	7	0	※	※	※	※	※	※	※
Abdelraouf A 2015	prospective cohort	6	0	※	※	※	※	0	※	※
Soto E 2022	retrospective cohort	7	※	※	※	※	※※	※	0	0
Borham MM 2014	prospective cohort	7	0	※	※	※	※	※	※	※
Milano CA 1999	retrospective cohort	7	0	※	※	※	※	※	※	※
Bhat JA 2020	retrospective cohort	7	0	※	※	※	※	※	※	※
Chaudhry A 2019	retrospective cohort	8	※	※	※	※	※	※	※	※
Blok RD 2019	retrospective cohort	7	0	※	※	※	※※	※	※	0
Ozben V 2016	retrospective cohort	8	※	※	※	※	※※	※	0	※
Rosso E 2012	retrospective cohort	8	0	※	※	※	※	※	0	※
Marzouk M 2021	retrospective cohort	7	0	※	※	※	※	※	※	※
Tewarie L 2019	retrospective cohort	6	0	※	※	※	※	※	0	※
Lale A 2020	retrospective cohort	7	※	※	※	※	※	※	0	※
Aukema TS 2009	retrospective cohort	7	※	※	※	※	0	※	※	※
Tsaroucha AK 2005	retrospective cohort	5	0	※	※	※	※	※	0	0
Pechlivanides G 1991	retrospective cohort	7	※	※	※	※	0	※	※	※
Ozben V 2018	retrospective cohort	7	※	※	※	※	※	※	0	※
Kayaalp C 2002	prospective cohort	5	0	※	※	※	※	0	0	※
Slaman AE 2022	prospective cohort	8	※	※	※	※	※	※	※	※
John H 1991	retrospective cohort	7	0	※	※	※	※	※	※	※

Abd Ellatif ME 2013	retrospective cohort	7	※	※	※	※	※	※	0	※
Lin BC 2017	retrospective cohort	7	※	※	※	※	※	※	0	※
Lo HC 2011	retrospective cohort	7	※	※	※	※	※	※	0	※
Abdallah E 2020	retrospective cohort	9	※	※	※	※	※※	※	※	※
Kim MG 2015	retrospective cohort	7	※	※	※	※	0	※	※	※
Zaouche A 2001	retrospective cohort	7	0	※	※	※	※	※	※	※
Ozacmak ID 2000	prospective cohort	7	0	※	※	※	※	※	※	※
Ye P 2016	retrospective cohort	7	※	※	※	※	※	※	0	※
Kouraklis G 2005	retrospective cohort	7	0	※	※	※	※	※	※	※
Hamamci EO 2005	retrospective cohort	6	0	※	※	※	0	※	※	※
Miyamoto Y 2016	retrospective cohort	6	0	※	※	※	※	※	0	※
Panaro F 2014	prospective cohort	7	※	※	※	※	※	※	0	※
Okano K 2013	retrospective cohort	6	0	※	※	※	※	※	0	※
Welten VM 2019	retrospective cohort	7	※	※	※	※	※	※	0	※
Shah OJ 2015	retrospective cohort	7	※	※	※	※	※	※	0	※
Husain M 2011	retrospective cohort	7	※	※	※	※	※	※	0	※
Sepesi B 2012	retrospective cohort	8	※	※	※	※	※	※	※	※
AlHaddad M 2021	retrospective cohort	8	※	※	※	※	※	※	※	※
Naga MA 2020	retrospective cohort	8	※	※	※	※	※	※	※	※
Ölmez A 2019	retrospective cohort	8	※	※	※	※	※※	※	0	※
López-Monjardin H 1998	retrospective cohort	6	0	※	※	※	※	※	0	※
Takatsuki M 2021	retrospective cohort	8	※	※	※	※	※	※	※	※
Matsuda H 2012	retrospective cohort	6	※	※	※	※	0	※	0	※
Blok RD 2019 (P)	retrospective cohort	8	※	※	※	※	※	※	※	※
Hashimoto I 2014	retrospective cohort	5	0	※	※	※	0	※	0	※
Pan CW 2020	retrospective cohort	6	0	※	※	※	※	※	0	※
Zhou Y 2019	retrospective cohort	7	0	※	※	※	※	※	※	※
Blok RD 2019	retrospective cohort	8	※	※	※	※	※	※	※	※
Gourgiotis S 2007	retrospective cohort	7	※	※	※	※	0	※	※	※
Erdener A 1992	retrospective cohort	6	0	※	※	※	0	※	※	※
Reza Mousavi S 2005	prospective cohort	7	0	※	※	※	※	※	※	※
Agaoglu N 2003	retrospective cohort	5	0	※	※	※	0	※	※	0
Xu S 2020	retrospective cohort	5	0	※	※	※	0	※	※	※

Sabry K 2018	retrospective cohort	7	※	※	※	※	0	※	※	※
Erdem E 1998	retrospective cohort	8	※	※	※	※	※	※	※	※
Smith D 2018	retrospective cohort	6	0	※	※	※	0	※	※	※
Muftuoglu MAT 2005	retrospective cohort	8	※	※	※	※	※	※	※	※
Manterola C 2013	prospective cohort	8	※	※	※	※	※	※	※	※
Tani M 2012	retrospective cohort	7	※	※	※	※	※	※	0	※
Nanashima A 2012	retrospective cohort	7	0	※	※	※	※	※	※	※
Nagata M 2020	retrospective cohort	6	0	※	※	※	※	※	0	※
Hultman CS 2010	retrospective cohort	7	0	※	※	※	※	※	※	※

Quality evaluation of included studies was conducted independently by two reviewers using the Cochrane risk of bias tool (ROB2) for randomized controlled trials or the Newcastle-Ottawa Scale (NOS) for nonrandomized studies.
