# Supplementary material for: The effect of omentoplasty in various surgical operations: systematic review and meta-analysis
Source: Int J Surg. 2024 Mar 4;110(6):3778–94. doi: 10.1097/JS9.0000000000001240 (PMC11175753; doi:10.1097/JS9.0000000000001240)
Supplement: Supplementary file 5 [file js9-110-3778-s006.pdf]

Table S3. Publication bias. The Egger's test of Stata software (Stata 16.0) was used to detect publication bias for the five surgical procedures with or without omentoplasty.

surgery	significant difference	P value of Egger's test
**1. Esophageal surgery (＜10 studies)**		
1.1 incidence of overall complications	yes	0.011
1.2 incidence of postoperative infection	no	0.511
1.3 incidence of anastomotic leakage	no	0.866
1.4 incidence of mortality	no	0.182
**2. Thoracic surgery (more than 10 studies)**		
2.1 incidence of overall complications(⩾10 studies)	no	0.538
2.2 incidence of postoperative infection	no	0.254
2.3 incidence of reoperation	yes	0.021
2.4 mortality	no	0.644
2.5 hospital stay	no	0.389
**3. Gastrointestinal surgery (≥10 studies)**		
3.1 incidence of overall complications(⩾10 studies)	no	0.56
3.2 incidence of postoperative bleeding(⩾10 studies)	yes	0.004
3.3 incidence of postoperative infection(⩾10 studies)	no	0.06
3.4 incidence of anastomotic leakage(⩾10 studies)	no	0.186
3.5 incidence of fistula	no	0.023
3.6 incidence of delayed gastrointestinal emptying	no	0.151
3.7 mortality(⩾10 studies)	no	0.555
3.8 hospital day	no	0.179
**4. Liver surgery (≥10 studies)**		
4.1 incidence of overall complications(⩾10 studies)	no	0.982
4.2 incidence of infection(⩾10 studies)	yes	0.045
4.3 incidence of anastomotic leakage	no	0.215
4.4 incidence of fistula(⩾10 studies)	no	0.111
4.5 incidence of recurrence	no	0.595
4.6 mortality	no	0.941
4.7 hospital stay	no	0.066
**5. Pelvi-perineal surgery (≥10 studies)**		
5.1 incidence of overall complication(⩾10 studies)	no	0.07
5.2 incidence of bleeding	no	0.208
5.3 incidence of infection	no	0.376
5.4 incidence of wound dehiscence	no	0.914
5.5 incidence of anastomotic leak	no	0.187
5.6 incidence of ileus(⩾10 studies)	no	0.463
5.7 incidence of reoperation	no	0.084
5.8 mortality	no	0.203
