# Supplementary material for: The effect of omentoplasty in various surgical operations: systematic review and meta-analysis
Source: Int J Surg. 2024 Mar 4;110(6):3778–94. doi: 10.1097/JS9.0000000000001240 (PMC11175753; doi:10.1097/JS9.0000000000001240)

Supplementary Figure 1: Publication bias analyses

1. Esophageal surgery

1.1 incidence of overall complications

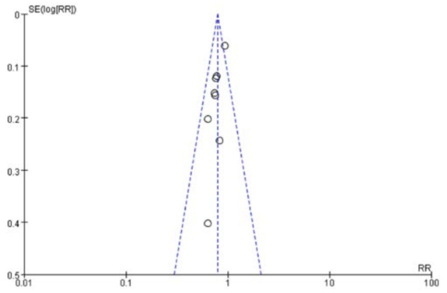

1.2 incidence of postoperative infection

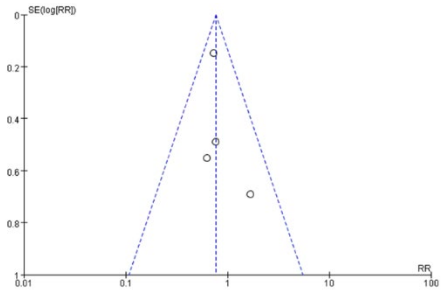

1.3 incidence of anastomotic leakage

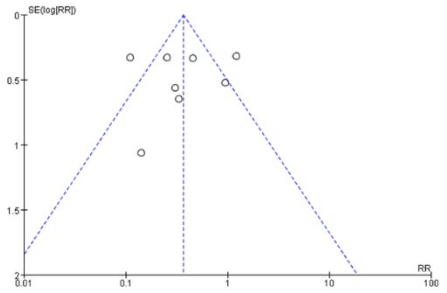

1.4 incidence of mortality

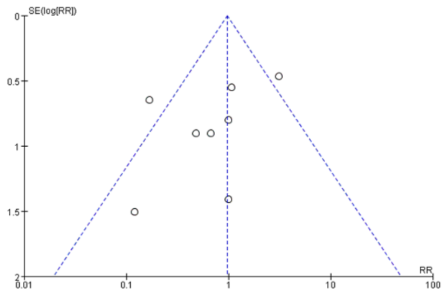

2.Tharocic surgery

2.1 incidence of overall complications

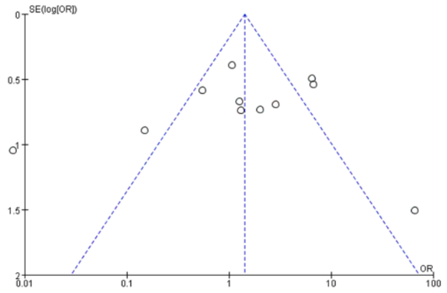

2.2 incidence of postoperative infection

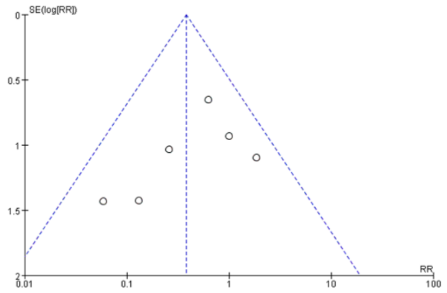

2.3 incidence of reoperation

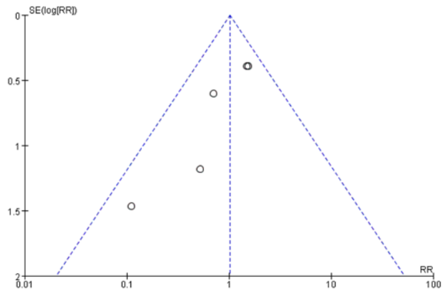

2.4 mortality

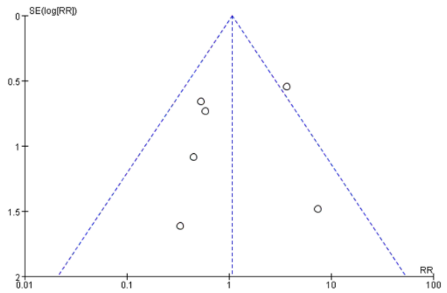

2.5 hospital stay

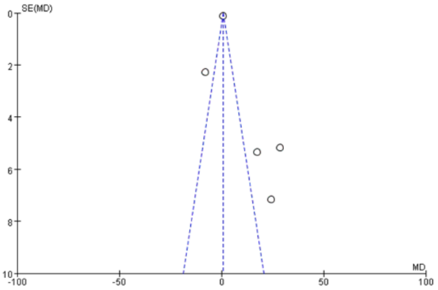

3. Gastrointestinal surgery

3.1 incidence of overall complications

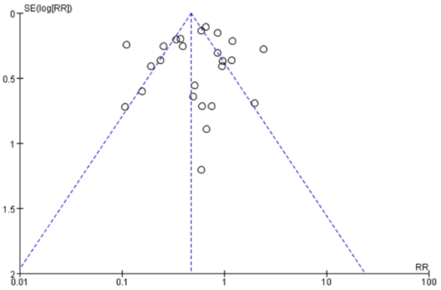

3.2 incidence of postoperative bleeding

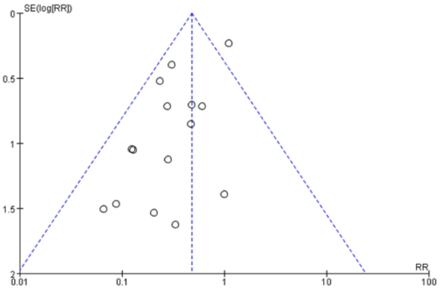

3.3 incidence of postoperative infection

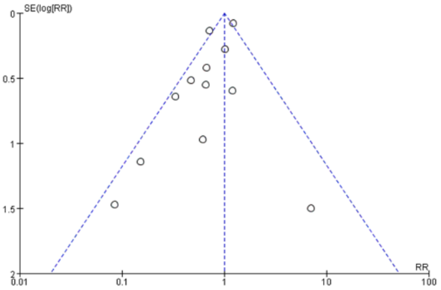

3.4 incidence of anastomotic leakage

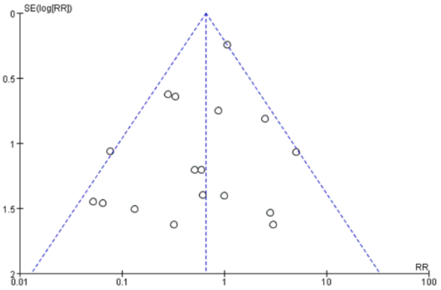

3.5 incidence of fistula

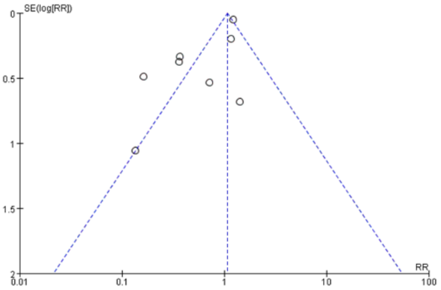

3.6 incidence of delayed gastrointestinal emptying

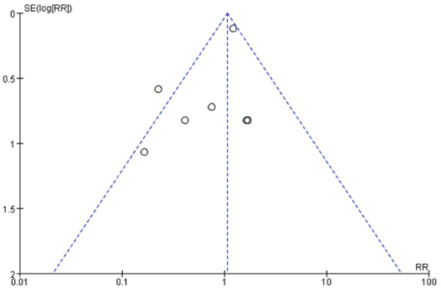

3.7 mortality

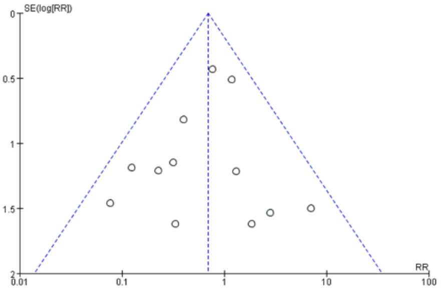

3.8 hospital day

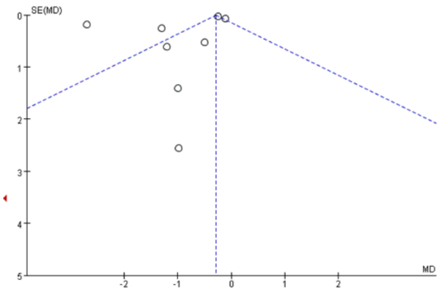

4. Liver sugery

4.1 incidence of overall complications

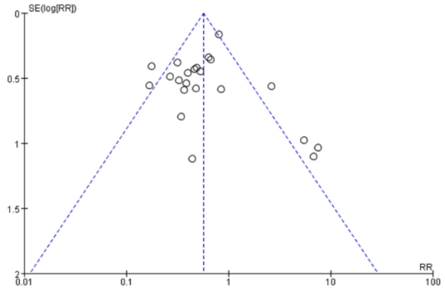

4.2 incidence of infection

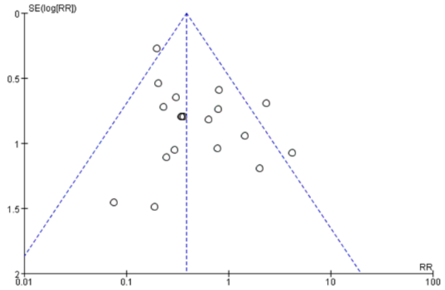

4.3 incidence of anastomotic leakage

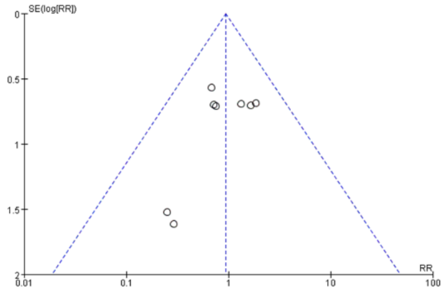

4.4 incidence of fistula

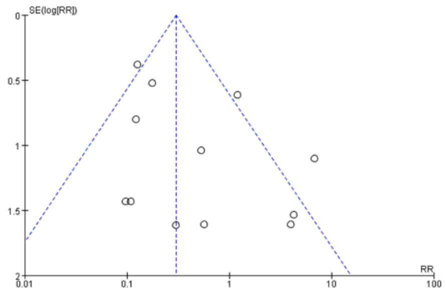

4.5 incidence of recurrence

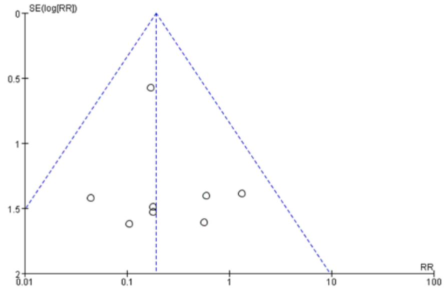

4.6 mortality

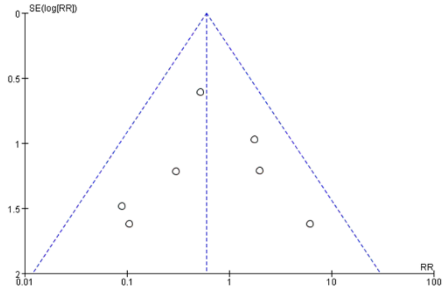

4.7 hospital stay

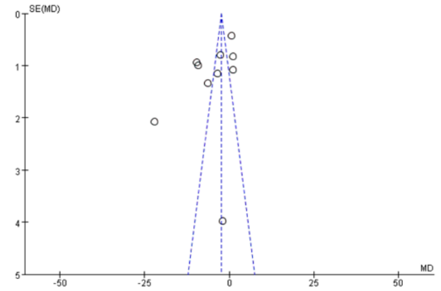

5. Pelvi-perineal surgery

5.1 incidence of overall complication

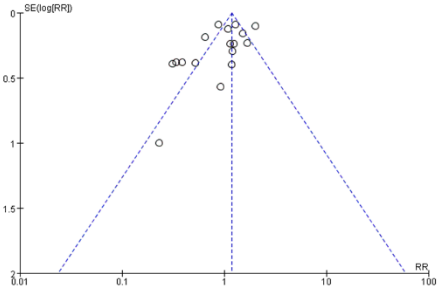

5.2 incidence of bleeding

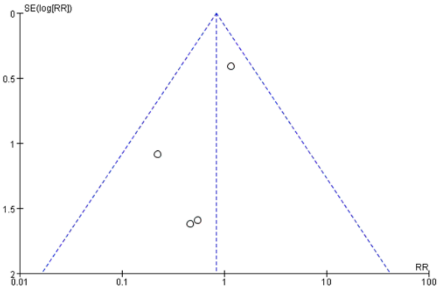

5.3 incidence of infection

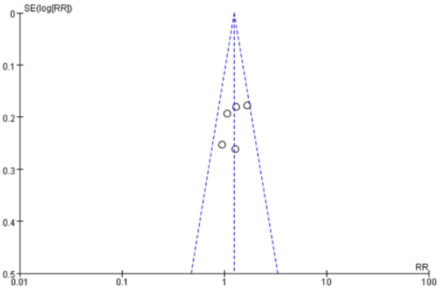

5.4 incidence of wound dehiscence

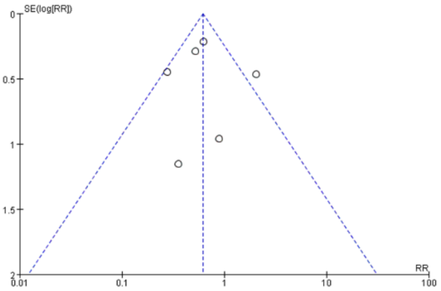

5.5 incidence of anastomotic leakage

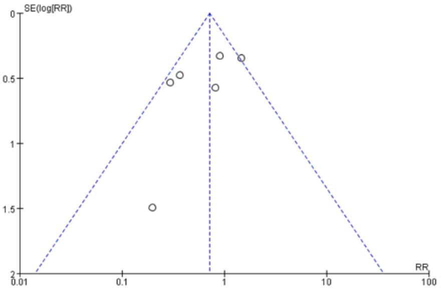

5.6 incidence of ileus

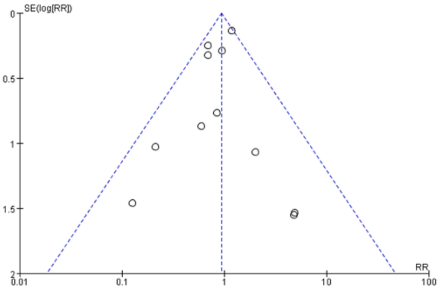

5.7 incidence of reoperation

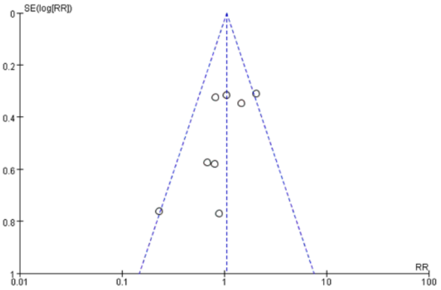

5.8 mortality

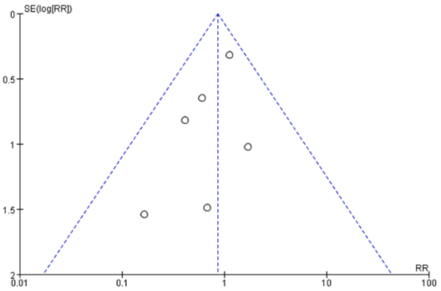

Supplement: Supplementary file 6 [file js9-110-3778-s007.pdf]
