# Supplementary material for: The effect of omentoplasty in various surgical operations: systematic review and meta-analysis
Source: Int J Surg. 2024 Mar 4;110(6):3778–94. doi: 10.1097/JS9.0000000000001240 (PMC11175753; doi:10.1097/JS9.0000000000001240)
Supplement: Supplementary file 7 [file js9-110-3778-s008.pdf]

Table S4. Meta-regression analysis. For their extremely heterogeneous results (I2 > 90%), hospital stays in gastrointestinal, liver, and thoracic surgery underwent meta-regression analysis using Stata 16. These analyses screened three potential influential factors (study type, developed or developing region, study period). 1=omentoplasty group; 2=non-omentoplasty group; n=number of patients; mean=mean difference; sd= standard difference.

A Gastrointestinal sugery

study	n1	mean1	sd1	n2	mean2	sd2	studytype	region	studyperiod
Abd Ellatif ME 2013	108	5	3.2	71	5.5	3.6	0	0	0
Abosayed AK 2022	45	1.02	0.15	46	1.13	0.5	1	0	1
AlHaddad M 2021	70	1.8	0.97	70	4.5	1.2	0	0	1
Kim MG 2015	56	10.2	6.8	14	17.8	12.7	0	1	0
Pan CW 2020	49	11.59	10.52	30	12.57	11.32	0	0	1
Pilone V 2019	96	4.5	1.5	90	5.8	2	1	1	1
Rosso E 2012	33	16	6	28	17	5	0	1	0
Sabry K 2018	1000	1	0.5	1000	1.25	0.75	0	0	1
Shah OJ 2015	101	8.3	2.8	46	9.5	3.7	0	0	0
							1: RCT	1: developed	1: ≥2017
							0: cohort	0: developing	0: < 2017
							P=0.524	P=0.545	P= 0.944

B Liver surgery

study	n1	mean1	sd1	n2	mean2	sd2	studytype	region	studyperiod
Agaoglu N 2003	16	12	2.2	28	15.5	5.4	0	0	0
Bhat JA 2020	30	8.7	2	27	18.4	4.5	0	0	1
Borham MM 2014	32	6.5	1.9	28	15.6	5	0	0	1
Hamamci EO 2005	16	7	3.8	23	6	2.5	0	0	0
Manterola C 2013	48	6.2	3.8	40	5.2	3.9	0	0	1
Nanashima A 2012	14	33	12	65	35	19	0	1	0
Okano K 2013	25	25	4.79	25	31.4	4.69	0	1	1
Pechlivanides G	95	15	9	57	37	14	0	1	0
Wani AA 2013	22	6.95	1.98	28	9.6	3.58	1	0	1
Xu S 2020	25	5.08	1.63	24	4.42	1.44	0	0	1
							1: RCT	1: developed	1: ≥2013
							0: cohort	0: developing	0: < 2013
							P=0.717	P=0.139	P= 0.639

C Thoracic surgery

study	n1	mean1	sd1	n2	mean2	sd2	studytype	region	studyperiod
Barnea Y 2000	15	32.06	14.67	15	14.6	14.59	1	1	0
El-Sherpiny WY	24	5	0.43	20	4.17	0.39	1	0	1
Marzouk M 2021	33	58.9	40.3	364	34.6	27.4	0	1	1
Milano CA 1999	21	10.7	5.5	38	18.8	12	0	1	0
Tewarie L 2019	19	53.8	19	20	25.2	12.4	0	1	1
							1: RCT/clinical trial	1: developed	1: ≥2012
							0: cohort	0: developing	0: < 2012
							P=0.764	P= 0.502	P= 0.451
