# Supplementary material for: The effect of omentoplasty in various surgical operations: systematic review and meta-analysis
Source: Int J Surg. 2024 Mar 4;110(6):3778–94. doi: 10.1097/JS9.0000000000001240 (PMC11175753; doi:10.1097/JS9.0000000000001240)
Supplement: Supplementary file 8 [file js9-110-3778-s009.pdf]

Table S5. Sensitivity analyses. Sensitivity analyses were performed by omitting each study individually for each postoperative outcome with Stata 16. The remark section showed the specific literature that caused significant differences after omission.

surgery	significant difference after omission	original 95%CI	remark
**1. Esophageal surgery**			
1.1 incidence of overall complications	no		
1.2 incidence of postoperative infection	yes	[0.58-1.01]	The results were significant after Zhou D 2018 was excluded
1.3 incidence of anastomotic leakage	no		
1.4 incidence of mortality	no		
**2. Thoracic surgery**			
2.1 incidence of overall complications	no		
2.2 incidence of postoperative infection	yes	[0.18-0.78]	The results were not significant after Zhou Y 2019 was excluded
2.3 incidence of reoperation	no		
2.4 mortality	no		
2.5 hospital stay	yes	[1.07-20.84]	Results were not significant after exclusion by Barnea Y 2000, El-Sherpiny WY 2021, Marzouk M 2021 or Tewarie L 2019
**3. Gastrointestinal surgery**			
3.1 incidence of overall complications	no		
3.2 incidence of postoperative bleeding	no		
3.3 incidence of postoperative infection	yes	[0.56-1.08]	The results were significant after Tani M 2012 exclusion
3.4 incidence of anastomotic leakage	no		
3.5 incidence of fistula	yes	[0.34-1.00]	Results were significant after exclusion by Rosso E 2012 or Tani M 2012
3.6 incidence of delayed gastrointestinal emptying	no		
3.7 mortality	yes	[0.44-1.09]	The results were significant after Negm S 2022 exclusion
3.8 hospital day	no		
**4. Liver surgery**			
4.1 incidence of overall complications	no		
4.2 Incidence of infection	no		
4.3 incidence of anastomotic leakage	no		
4.4 incidence of fistula	yes	[0.18-0.99]	Results were not significant after exclusion of Agaoglu N 2003, Bhat JA 2020, Gourgiotis S 2007, Hamamci EO 2005, Kayaalp C 2002, Muftuoglu MAT 2005, Ozacmak ID 2000 or Pechlivanides G1991
4.5 incidence of recurrence	no		
4.6 mortality	no		
4.7 hospital stay	no		
**5. Pelvi-perineal surgery**			
5.1 incidence of overall complication	no		

5.2	incidence of bleeding	no		
5.3	incidence of infection	no		
5.4	incidence of wound dehiscence	yes	[0.38-1.05]	The results were significant after exclusion of Welten VM 2019
5.5	incidence of anastomotic leak	yes	[0.38-1.19]	The results were significant after Ozben V 2016 was removed
5.6	incidence of ileus	no		
5.7	incidence of reoperation	no		
5.8	mortality	no		
